# Global effects of local food-production crises: a virtual water perspective

**DOI:** 10.1038/srep18803

**Published:** 2016-01-25

**Authors:** Stefania Tamea, Francesco Laio, Luca Ridolfi

**Affiliations:** 1 Politecnico di Torino, Department of Environment, Land and Infrastructure Engineering, Turin, 10129, Italy

## Abstract

By importing food and agricultural goods, countries cope with the heterogeneous global water distribution and often rely on water resources available abroad. The virtual displacement of the water used to produce such goods (known as *virtual water*) connects together, in a global water system, all countries participating to the international trade network. Local food-production crises, having social, economic or environmental origin, propagate in this network, modifying the virtual water trade and perturbing local and global food availability, quantified in terms of virtual water. We analyze here the possible effects of local crises by developing a new propagation model, parsimonious but grounded on data-based and statistically-verified assumptions, whose effectiveness is proved on the Argentinean crisis in 2008–09. The model serves as the basis to propose indicators of crisis impact and country vulnerability to external food-production crises, which highlight that countries with largest water resources have the highest impact on the international trade, and that not only water-scarce but also wealthy and globalized countries are among the most vulnerable to external crises. The temporal analysis reveals that global average vulnerability has increased over time and that stronger effects of crises are now found in countries with low food (and water) availability.

The international trade of agricultural goods causes a virtual displacement of the water used to produce such goods, “embedded” in trade as a factor of production^1^,^2^. The volume of freshwater (rainfall, surface water and groundwater) used for the production of such goods, and no more available for other uses at the production site, is known as virtual water (VW). Volumes can be summed across all different commodities and represent an alternative quantity to money or calories to investigate different aspects of the international trade. Agricultural commodities are by far, the most water-intensive traded products^3^ and they are in large part ultimately devoted to human consumption. Virtual water thus provides a quantitative general framework for the investigation of the water-food-trade nexus, posing the emphasis on water resources and the environment^4^.

Virtual water exchanged between producing and consuming countries defines flows across an international network associated to the commodity trade. This *virtual water trade* has relevant environmental and socio-economic implications, the most important one being that water-scarce countries can rely on water resources available abroad. In fact, in semi-arid countries, water scarcity limits the local agricultural production posing a key challenge to food security^5^. Importing food produced elsewhere enables such countries to overtake water resource limitations and to increase the food available to the population^6^, while possibly saving global water resources^7^,^8^. As a drawback, VW trade results in a marked country interdependency, and possibly in harsh inequalities when economic advantages, rather than environmental ones, drive the exchange of goods^9^,^10^.

In recent years, the international VW trade underwent marked modifications, following the expansion and evolution of commodity trade^11^,^12^,^13^. In the period 1996–2005, the average volume of virtual water exchanged worldwide was 2320 km^3^ per year and represented about one fifth of the total volume embedded in consumed goods^14^. From 1986 to 2010, the traded VW volume grew more than twofold and the number of trade links had a widespread increase^15^, determining an evolution towards a globalized water system^15^,^16^,^17^. Given the increasing country interdependency, concerns have been raised about the sustainability of population growth under the constrain of limited global water resources^18^ and the potential reduction of societal resilience to crises^19^.

Globalization of commodity trade contributes to improve food availability and to sustain population by reducing the dependence on local resources^20^,^21^,^22^. In contrast, globalization favors the propagation of crises, as it leads to interconnected production-consumption systems that are complex and vulnerable to failures^23^. Models for the propagation of crises, or contagion, in networks have been developed in the context of complex systems, mostly basing on the random graphs theory^24^. By this approach, several studies about financial networks have addressed the risk associated to default events and how the spread of a crisis depends crucially on the pattern of interconnectedness between network nodes^25^,^26^,^27^. The structure of network interdependencies and feedbacks may also generate amplified responses to shocks, for example in input-output production networks^28^,^29^. However, the international trade network, of both food commodities and VW, is complex and its features and dynamics cannot be well described by random graphs^30^. Given the recognition that, also in international trade, network topology plays a key role in the spreading of economic crises^31^, specific models of shock propagation and vulnerability measures need to be developed for the (VW) trade network^32^,^33^.

A different approach to the analysis of trade dynamics, with specific reference to its effects on water endowments, is represented by agro-economic models, such as GTAP-W^34^ or IMPACT^35^. These models are based on the general equilibrium theory and mimic the dynamics of trade and associated use of water resources by also considering market mechanisms and price variability. Models based on the general equilibrium theory typically lend themselves to scenario analyses, providing qualitative indications about the future behavior of the global water system; in contrast, their coarse spatial and sectorial resolution and large data requirements limits their use for the quantitative analysis of the propagation of local crises, which is the aim of the present paper.

Pursuing a different approach, we propose here an analysis of the effects of local food-production crises at the annual scale, which bases on a simple and novel mechanistic model. Building on data-based rules, statistically verified assumptions and very few parameters, the model mimics the propagation of crises taking advantage of the observed network structure and VW flows. The model is then tested on a real case to confirm the correctness of the propagation mechanism, and it is then applied to the assessment of countries’ impact and vulnerability to local crises.

## Modeling the effect of crises

A national annual virtual water balance, following from a food balance expressed in terms of virtual water equivalent, can be stated for each country, *c*, at any year, 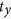, as


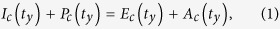


where 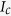 and 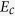 are the virtual water volumes associated to the import and export of agricultural commodities of country *c*, respectively; 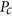 is the virtual water embedded in the primary agricultural production (or *water footprint of production*), and 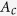 is the remaining term for the balance closure (i.e., the water footprint of food supply and stock). The term 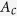 represents the virtual water embedded in the annual *internal availability* of agricultural goods which includes food consumption, stock variation and other minor terms, such as waste. A decrease of 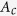 may indicate a decreased consumption of agricultural goods or a reduction of their stocks, indifferently, as separating these components or mimicking stock dynamics goes beyond the scope of the present work.

A crisis is identified by a marked decrease of agricultural production, expressed in terms of equivalent virtual water volume. This reduction can be triggered by environmental, social or economic events such as droughts, floods, pests, conflicts or economic crises, disregarding the few cases in which the decrease in agricultural production is counterpoised by an increased use of water resources. When a country undergoes a local crisis, the VW embedded in its agricultural production is decreased by a fraction, *β*, of the initial water footprint of production. The VW loss induced by the crisis is partitioned into a reduction of internal availability and a reduction of export, according to the relative weight of the two terms in the country VW balance (1). The country-specific export weight, a, identifies the so-called *export propensity* of the country.

The reduction of export propagates to all direct trading partners proportionally to the initial VW flows towards each of them, and all trading partners find themselves with a reduced volume of VW in the balance. The rule of proportional propagation is strongly supported by data, as detailed in the Method section. The reduced volume is again partitioned between internal availability and export in each country and propagated towards their trading partners. Calculations proceed iteratively until a prescribed level of accuracy is reached. At the end of the simulation, the reduction in production occurred in the first country reflects into modified VW flows in the trade network and reductions of virtual water availability worldwide, with a geographic distribution that depends on the network structure (topology and node-to-node trade). The proposed propagation model is similar to the one developed by Lee **et al.**^31^ for the macroeconomic trade network, but here we use country-specific behaviors and data-based export propensities.

The model bases on assumptions that are statistically validated on real data of VW embedded in food production and trade (see the Method section): in particular, we found that (i) a decrease of agricultural production in a country is associated to a decrease of export in the same country, (ii) a decrease of import is associated to a decrease of export, and (iii) a decrease of production does not generally imply an increase of import. There is no clear evidence, from our dataset, of compensatory mechanisms by which countries suffering a reduction of VW import from one partner increase the VW import from elsewhere (new or existing links), nor that occasional compensation is related to country wealth. The use of global rules and local coefficients represents a compromise which, although not considering some specific policies (e.g., modification of imports, activation of new trade links, substitution of commodities, role of commodity prices), allows one to mimic the core dynamics of crisis propagation. The use of annual data does not enable the investigation of sub-annual consequences of food-production crisis, but the propagation model could be adapted to different time scales if suitable data were available.

## Results

### Test case: the Argentinean crisis

In 2008–09 Argentina underwent a crisis which markedly affected its economy and agricultural production. Triggered by the international financial crisis and by the local political economy (e.g., taxation of agricultural exports^36^,^37^), which induced a major reduction of exports, a decreased agricultural production occurred, strengthened by a prolonged drought within the country^38^. The decrease of Argentinean exports replicated that of agricultural production (see Supplementary Material, Figure S1) as the VW volumes of export and production are highly correlated (correlation coefficient equal to 0.979). The overall decrease of VW embedded in agricultural production and export (from 2007 to 2009) amounted for both variables to 25.8%.

**Figure 1:**
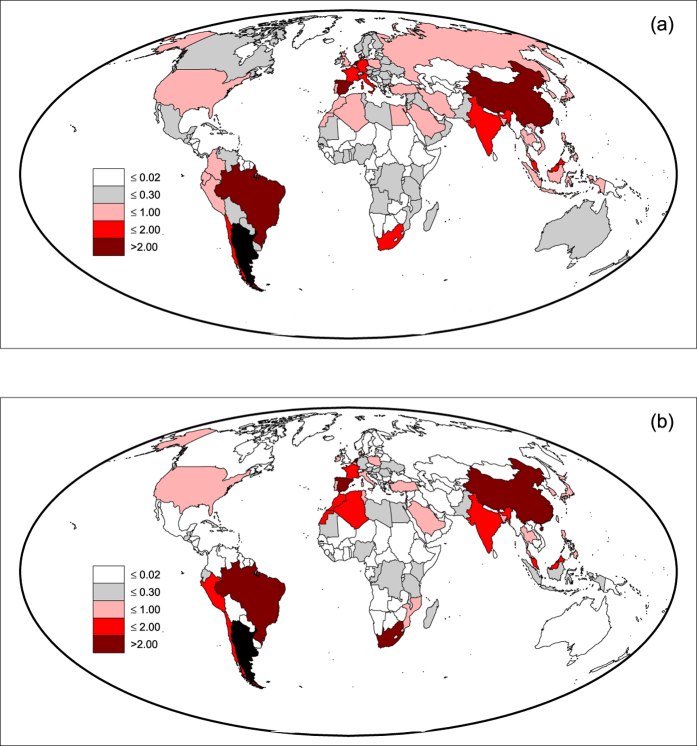
Effects of the crisis in Argentina in 2008. (**a**) Variations of VW import of countries, as obtained from the model, and (**b**) variations of VW imports from Argentina between 2007 and 2009 as from data; in both panels, positive variations indicate a decrease of VW import, measured in km^3^ [maps created with Matlab^®^ R14 software, Mapping Toolbox v.2.0.3 (http://uk.mathworks.com/products/mapping/)].

The two-year decrease, corresponding to a parameter value 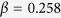, is used to run the propagation model for the Argentinean case study, starting from the unperturbed VW trade network in 2007. The impact of the Argentinean crisis on the VW trade resulting from the model simulation is shown in Fig. 1a, mapping the variations of VW import induced in all countries by the crisis. This is compared, in Fig. 1b, to the difference observed from 2007 to 2009 (final year of the crisis) in the VW fluxes originated from Argentina. This choice allows us to isolate the effect of the Argentinean crisis from the manifold modifications of overall VW trade induced by other political-economic dynamics.

The model results compare very well with data. Most impacted countries are correctly identified by the model and are Brazil, China and Spain, where the VW fluxes have decreased by more than 2 km^3^ (from 30 to 50% of initial VW fluxes). Marked impacts are also suffered by South Africa, India, Malaysia, France and Chile, with a real and modeled decrease of 1–2 km^3^, again corresponding to 30–50% of initial VW fluxes. Also countries with intermediate and negligible variations of VW trade show a good matching with the model results. The marked decrease of import observed in Morocco and Algeria is likely due to the initial phase of the Arab Spring rather than to the Argentinean crisis^39^, and are thus overlooked by the model.

### Propagation of crises

**Figure 2:**
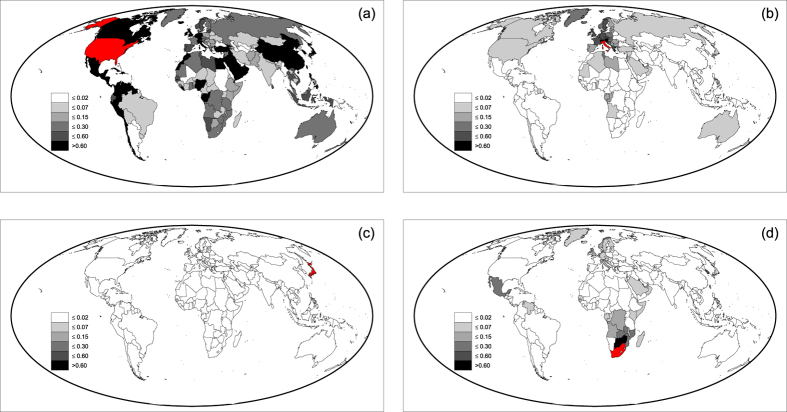
Effect of possible crises originated in the USA (**a**), Italy (**b**), Japan (**c**), and South Africa (**d**) in 2011 (*β* = 0.3), quantified by the percentage variations of per capita VW availability in all other countries. Dark grey indicates stronger impacts and light grey smaller impacts; red color identifies countries where the crises originated from [maps created with Matlab^®^ software, Mapping Toolbox v.2.0.3 (http://uk.mathworks.com/products/mapping/)].

Having shown the capability of the proposed model to mimic real dynamics, we use it to infer the propagation of possible crises hitting four different countries: USA, Italy, Japan and South Africa. Crises are quantified by a decrease of 30% of the water footprint of agricultural production in 2011, 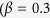, see the Method section), then propagated in the virtual water trade network depicted by the 2011 data. Results are illustrated in Fig. 2 and Table 1.

The most impacted country in these crises is not necessarily the one receiving the largest share of export from the hit country. In fact, the model mimics the cascade dynamics on the network and reproduces the superimposition of disturbances reaching a country directly and indirectly, through different trade connections. This leads, for example, to the case of Japan, whose major trading partner in 2011 was Hong Kong, but the country modeled as having the overall largest reduction of import is China (−24 Mm^3^ of VW); the same also occurs to possible crises hitting some large exporting countries, such as France and Indonesia, as well as minor countries (crises not shown).

The geographic extension of the four possible crises and the strength of impacts in all countries, measured by the percentage variation (decrease) of the per-capita VW internal availability are highlighted in Fig. 2. Variations of per-capita VW availability are small (see Table 1) because traded goods represent only a fraction of global agricultural production and because the crisis is spread within the network, distributing its effects on several countries. Having small variations is also coherent with the fact of considering a fixed network structure during crises propagation and only modeling fluxes variations; crises disrupting the trade network are not considered in this modeling framework.

Marked differences in the results are due to the different hit strengths, in that greater VW losses occur in hit countries with greater agricultural production; however, the spreading of the crisis depends on the (weighted) network of country connections and the characteristics of the hit node. In fact, although Italy and South Africa undergo a similar VW loss, the resulting picture of highly impacted countries (dark grey) is quite different and ranges from most part of Europe in the case of Italy, to only few African countries in the case of South Africa. Also the extension of the crisis propagation is different and affects (with a variation greater than 0.1%) 54 countries with 510 total million people in the case of Italy, and 21 countries with 292 total million people in the case of South Africa.

Comparing the four possible crises, the network-based differences among hit nodes should be taken into account. Hit-node characteristics can be described, for example, by the sum of incoming and outgoing links (*unweighted degree*), by the sum of incoming and outgoing VW fluxes (*weighted degree*), and by the *eigenvector centrality*, i.e. an influence score dependent on the links and fluxes of connected nodes^40^ (see Table 1). The large impact of the USA can be explained by its large degree and eigenvector centrality, and the small impact of Japan is anticipated by the limited connectivity and peripheral position of the country, given its very low eigenvector centrality. Italy shows a similar weighted degree to Japan but its higher unweighted degree and eigenvector centrality lead to a much different crisis propagation and a wider distribution among many (European) large partners. The case of South Africa differentiates from Italy in that effects of the crisis are concentrated into very few countries with marked variations, while other connections are poorly affected; this can be anticipated by the low eigenvector centrality (ratio of 1:4 with Italy) which indicates a small influence of the node in the weighted network.

Following these considerations, it can be argued that information on the network structure and weights are relevant to identify macro features, such as crisis entity and extension, but the country-specific distribution of impacts requires a model of crisis propagation, capable of describing the multiple effects of the propagating crisis. This is what our proposed model does.

### Country impacts

**Figure 3:**
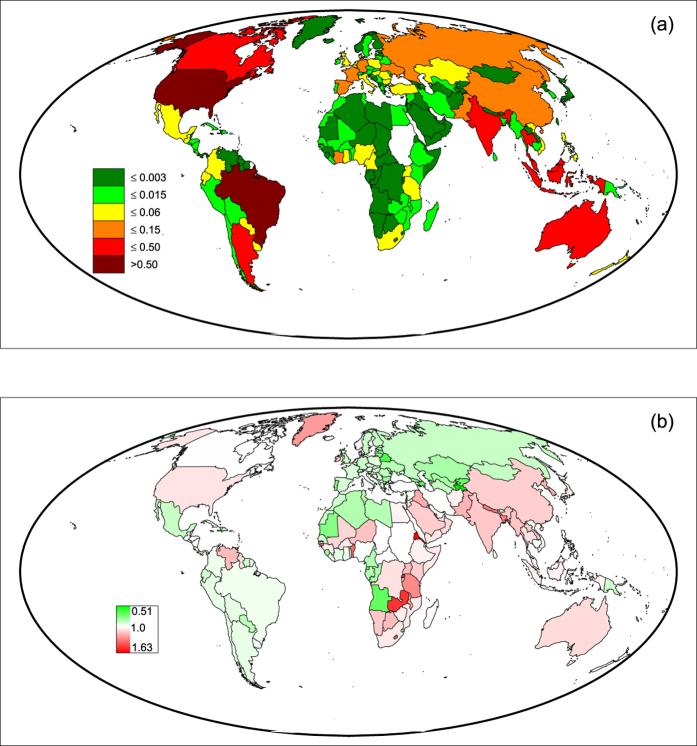
Absolute (**a**) and normalized (**b**) impact of countries in 2011 (*β* = 0.3), measured by the population-weighted average percentage reduction of VW availability induced in the rest of the world. White countries in panel (**a**) have missing data in 2011 [maps created with Matlab^®^ R14 software, Mapping Toolbox v.2.0.3 (http://uk.mathworks.com/products/mapping/)].

The absolute impact of a crisis can be measured by the population-weighted average percentage reduction of VW availability induced in the network, i.e.


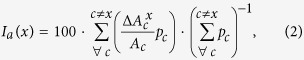


where 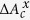 is the variation of VW availability in country *c*, having population 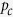, due to the crisis in country *x*. Considering equivalent crises in all countries (same *β*), one can compare the different impacts of countries on the network, as shown in Fig. 3a.

A strong correlation emerges between the absolute impact and the VW export fluxes of countries. In fact, large exporters of VW, such as the USA and Brazil have the largest impact on the network, European countries have an intermediate impact, while African and Middle Eastern countries have the smallest one. This correlation confirms the intuitive fact that crises with the largest impact are those hitting countries with larger VW fluxes towards the network. However, also the agricultural production of the hit country, *P*_*x*_, plays a role, in that VW volumes losses are defined as a fraction of it. To overcome such dependency on the crises entity and to highlight the role of the network, which is otherwise partially masked by the VW export of the hit country, a different indicator is required.

A normalized impact, 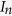, can thus be introduced, equal to the ratio between 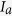 and the average percentage decrease of VW availability worldwide, evaluated as if the VW loss were uniformly distributed among the population, i.e.


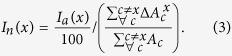


This normalized impact does not depend on the parameter *β* and it is invariant with the entity of the crises, thus enabling a more even comparison among different crises and countries.

A unit value of 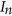 indicates a crisis inducing variations of VW availability that are *proportional* to the existing VW availability of countries. On the contrary, values distant from the unit indicate the tendency of a crisis to generate modifications of VW availability that are more than (or less than) proportional to the existing VW availability of countries. Taking the VW availability as a proxy of the VW embedded in food consumption in a country (map in the Supplementary Material, Figure S2), the normalized impact can be considered a measure of “equity” of a country impact. In fact, 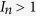 (red colors in Fig. 3b) identifies countries whose crises have stronger effects on low-consuming countries and can be considered *inequitable*, while 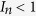 (green colors in Fig. 3b) identifies countries whose crises have stronger effects on high-consuming countries and are thus more *equitable*.

The joined analysis of absolute and normalized impacts in Fig. 3a,b allows us to make the following considerations. (i) Large American exporters, such as the USA, Canada, Brazil and Argentina, have a strong but equally distributed impact on the VW availability across the world. (ii) India, Thailand and Australia have a strong impact, which is mostly exerted on low-consuming countries; they are thus the most critical hotspots of the global water system. (iii) Intermediate-impact countries are usually unequal, with many of them affecting high-consuming countries (it is the case of major European countries, Russia, Ukraine, Mexico and Paraguay) and others affecting low-consuming countries (namely China, South Africa and Tanzania). (iv) Countries with low impact may show strong departure from the equal distribution of crises, however this is generally associated with small VW volumes traded and/or few trading partners, as can be seen, e.g., in Algeria or Zambia.

### Country vulnerability

In the assessment of the effects of local crises on the virtual water trade network, a key feature is the *vulnerability* of countries. This feature expresses the country exposure to food-production crises occurring elsewhere and propagating in the global trade network. To this purpose, we introduce a measure of vulnerability for the *c*-th country, computed as the percentage reduction of VW internal availability in *c* resulting from a crisis occurred in *x*. The measure is then cumulated over all crises occurring in the other countries, hit one at a time, that is


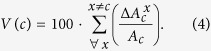


The vulnerability measure depends on the overall import of a country, on the network characteristics of source nodes (e.g., their export propensity) and on the country reliance on internal agricultural production. International economic dynamics are not accounted for in our evaluation of country vulnerability whereas the focus lies in the country dependency on food (and VW) supply through the trade network.

**Figure 4:**
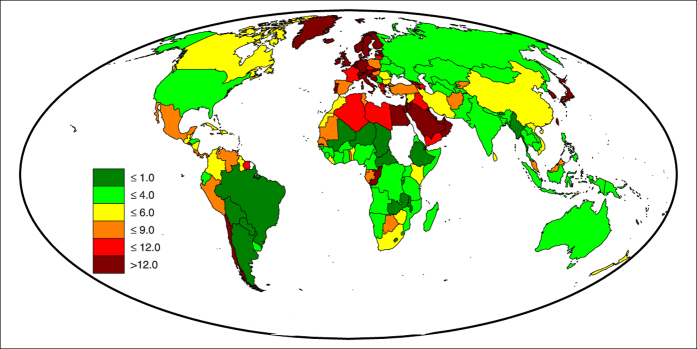
Vulnerability of countries in 2011 (*β* = 0.3), measured by the total percentage reduction of VW internal availability induced by all crises occurring in the rest of the world [map created with Matlab^®^ R14 software, Mapping Toolbox v.2.0.3 (http://uk.mathworks.com/products/mapping/)].

Country vulnerabilities are shown in Fig. 4 (details in the Supplementary Table) and highlight marked heterogeneities. Most vulnerable countries are found in Europe and in the Middle East and North-African (MENA) countries. In central European countries, the condition of vulnerability is dictated by the intense trading within the EU market. This community of countries is almost fully connected and exchanged VW volumes are large; thus the whole system responds to the crises and critical situations emerge when the superimposition of effects is analyzed. Scandinavian countries, instead, have limited VW trade and their vulnerability originates in the limited agricultural production and consequently low self-sufficiency (water footprint of production lower than VW availability). Within Europe, lower vulnerabilities are found only in countries such as Spain, France, and Poland, having a large agricultural production and low VW net import, thus a greater self-sufficiency in terms of (virtual) water resources.

In the MENA region, the large vulnerability is justified by the large VW import, a close second to Europe in per-capita terms^41^, and the limited VW export, although with a certain variability among countries. With large net import and low export propensity, most decreases of VW (thus food) volume directly impact the local availability rather than trading partners. This also happens to Japan and South Korea, which polarize such condition coupling large VW imports to negligible exports and forcing incoming crises to affect local VW availability. However, while in the latter countries the internal agricultural production and food stocks may play a role in mitigating the shortages and enable a better management of foreign crises, in the MENA region, where agricultural production suffers from water shortage, such mitigation cannot be pursued if not at high environmental and economic costs.

Other cases of medium-to-large vulnerabilities can be explained by large VW net imports despite the abundant agricultural production (such as Mexico and Turkey), or by low agricultural productions (in Suriname, Congo, Mali, Botswana), or a mix of large imports and low internal production (in Chile, Perù, Venezuela, Iran). Malaysia is a peculiar case, in that it shows large agricultural production and negative VW net import, suggesting a low vulnerability; however, its intense trading with large VW exporters such as Indonesia, India and Thailand, induces a great exposure to the risk of their failure, which in turn increases the overall vulnerability of the country.

Worldwide, low vulnerabilities (green colors) are found (i) in countries having large agricultural productions (e.g., Brazil, Argentina, USA, South Asia and Australia), which are self-sufficient in terms of virtual water (and food) even when external crises reach the country through trade, and (ii) in countries having low VW imports (e.g., African countries), which are peripheral in the VW trade network and thus not much “exposed” to external crises propagating in the VW trade network.

## Discussion

**Figure 5:**
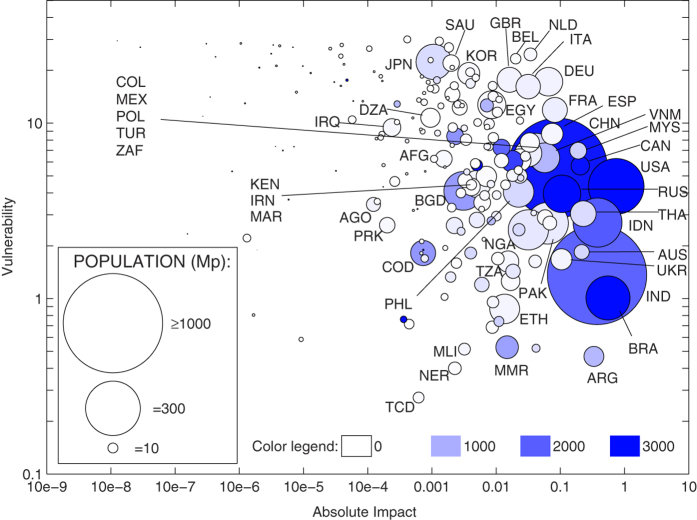
Absolute impact versus vulnerability of countries in 2011. Circle areas indicate country populations (in million people) and colors scale according to the total renewable water resources of countries (in km^3^/year). Numerical details are available in the Supplementary Table.

Quantitative metrics have been introduced to evaluate the impact and vulnerability of countries in the global water system, represented by the ensemble of national VW balances connected through the VW trade network^42^. We now present (Fig. 5) a joined analysis of the two metrics, including information on countries’s own water resources, measured by the total volume of actual renewable water resources, which summarizes the average annual flow of rivers and recharge of aquifers generated from endogenous precipitation and the incoming flow originating outside the country^43^.

In general, two key features emerge from Fig. 5: the large impact of water-rich countries (right part of the diagram), and the water scarcity of most vulnerable countries (top part of the diagram). Less populated countries have very small impact and highest vulnerability, suggesting a dependency on external resources, possibly due to lack of land or water. At the other extreme are the large producers of agricultural goods (USA, Brazil, India and Indonesia), having the highest impact and abundant freshwater resources but heterogeneous vulnerability, depending on their exposure to external crises through the VW import.

Central European countries have high impact and high vulnerability, due to their intense international trading. Agricultural production does not cover the internal VW demand, and these countries have to rely on foreign water resources, balancing trade openness and globalization with the exposure to external crises. Given that most of Europe is characterized by high agricultural efficiency, favorable climate and availability of freshwater resources, there is a potential for improving such condition. Pursuing an increase of agricultural production in these countries, although challenged by a sustainable land management (e.g., avoiding resource exploitation, loss of biodiversity/ecosystem services), would decrease the reliance on VW trade and lead to more affordable levels of vulnerability.

MENA countries are known to be markedly dependent on VW imports and, wherever local water resources are lacking, the vulnerability becomes very high (e.g., Saudi Arabia, Yemen, Algeria, Iraq). Egypt is one of the most vulnerable countries, due to its dependency of VW imports, but it also has non-negligible impact, thanks to its export of greenhouse and irrigated-agriculture products which, however, pose a marked pressure on its limited freshwater resources. In the case of MENA countries, vulnerability is dictated by water scarcity and can hardly be managed through agricultural policies and planning.

A high vulnerability is also found in Japan and South Korea, i.e., in countries dependent on the VW trade despite their water abundance, while at the other edge one finds Argentina, having a marked impact on the VW trade network, and a very low vulnerability although its water resources are not as abundant as for other large producers. The low vulnerability of Argentina is not disproved by the crisis in 2008 which, in fact, was not triggered by a decrease of food imports from other countries (see Supplementary Material, Figure S1); rather, it began with a decrease of Argentinean agricultural production, and subsequently spread into the trade network.

Among the countries with the largest impact on the global water system, critical situations emerge where crises are more likely. For example, countries having limited freshwater resources have a higher probability to undergo an environmental crisis or being negatively affected by a drought; among them, those having largest impact include (Fig. 5) Argentina, Thailand, Australia, Malaysia and Ukraine. On a different note, interpreting the above diagram through the country wealth, in terms of per-capita gross domestic product (Supplementary Material, Figure S3), it clearly emerges that richer countries are characterized by very high vulnerability to external crises. Moreover, if one considers less wealthy countries as having a higher propensity to socio-economic crises, it is important to highlight that India, Pakistan, Vietnam and Nigeria (to mention the largest ones) have low wealth but large impact on the VW trade network. In all these countries, the agricultural and water management as well as the political-economic efforts (local and international) should aim at preventing the occurrence and limiting the effects of crises, which might otherwise propagate affecting people and nations worldwide.

**Figure 6:**
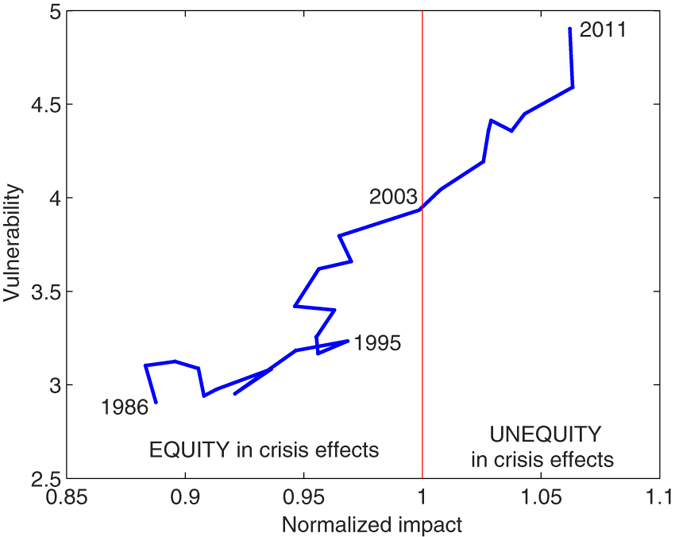
Temporal evolution, from 1986 to 2011, of world-averaged population-weighted values of vulnerability and normalized impact.

As a final remark, we analyze the water system globalization through the temporal evolution of world-scale indexes of impact and vulnerability, obtained as population-weighted averages (Fig. 6). The trajectory highlights an increase of both normalized impact and vulnerability at the global scale. At the beginning of the study period, the global water system favored *equitable* crises 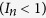, having stronger effects (i.e., more than proportional reductions of VW availability) on high-consuming countries. However, the system has changed in time, evolving towards a less equitable propagation of crises 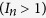, which imposes larger reductions on low-consuming countries. This evolution can be explained by the increase of VW import of less wealthy countries, which also increases their exposure to external crises, leading to a potential harshening of the inequality within the global water system at the occurrence of crises.

The world-average vulnerability also shows a clear evolution. It has been rather stable in the first decade of the study period, followed by a marked increase, only minimally affected by the world economical crisis in 2008–09. Driven by countries facing a fast growth, in population and wealth, the increase of vulnerability follows from the intensification of virtual water trade and the increase of global interconnectedness. On a country basis (Supplementary Figure S4) a decrease of vulnerability from 1986 to 2011 is observed in Brazil, Argentina and few other countries, while the dominant trend on the map is an increase, which is stronger in China and South-East of Asia, in the south of Africa, in Central America, in Turkey and Eastern Europe. The increase of vulnerability in the VW trade network, coupled to the decrease of equity in the effects of crises, confirms the complexity as well as the fragility of the global water system and calls for an increased attention on the problem of food-production crises.

## Methods

### Data

Data of virtual water trade are based on the commodity trade dataset of the Food and Agricultural Organization of the United Nations (Faostat)^44^. Detailed international trade matrices, 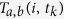, are available at the annual time scale from 1986 to 2011 and express the quantity of agricultural commodity *i* traded in year 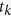 from country *a* to country *b*. Trade data are converted into virtual water flows by multiplying traded quantities by the crop virtual water content, 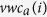, assuming that commodities are produced in the countries of origin of the flows. The total VW trade matrix, 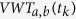, is then obtained by summing across all 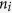 commodities, i.e.


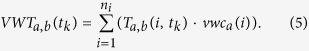


The considered crop virtual water content is the sum of green and blue water, i.e. rainfall and surface-/groundwater, contributing to crop evapotranspiration and data are provided by the global assessment of the Water Footprint Network^45^,^46^ as an average for the period 1996–2005. The centrality of such period with respect to our datasets grants the virtual water content to be reliable for our analysis, while variations due to interannual fluctuations are not accounted for.

Agricultural production data are available from the same Faostat database^44^ at the annual time scale for the period 1961–2011. Production data, 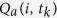, express the quantity of agricultural commodity *i* produced by country *a* in year 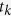; data are then converted into virtual water volumes using the country-specific and commodity-specific virtual water content^45^,^46^, including green and blue water. The overall production, to be included in the virtual water balance (1), results from the sum across all primary commodities, *m*_i_, in order to avoid for double-counting of water volumes involved in secondary products^47^, i.e.


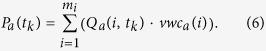


The Faostat database has also provided the population data for all countries over the period 1986–2011^44^, while the total volume of actual renewable water resources are given by the Aquastat database maintained by the Land and Water division of FAO^48^. The per-capita gross domestic product (GDP) at current prices in US Dollars is obtained from the national accounts section of the United Nations^49^. All data are available upon request to the corresponding author.

### The propagation model

The method bases on the virtual water balance of country *c* (Eq. (1)) where import, 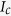, and export, 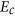, can be expressed through the virtual water trade matrix, *VWT*, as


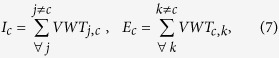


being 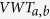 the virtual water flow from country *a* to country *b*. Knowledge of the virtual water embedded in production and trade (see the Data section) and choice of a prescribed year of data, enable the establishment of initial virtual water balances in all countries. The VW internal availability in each country is derived from Equation (1) and countries having negative availability are discarded from the analysis, as well as inactive countries without trade, for the impossibility to run the model in such cases (the number of remaining countries is 200–210, depending on the year, and discarded population is less than 1/1000 of world population). The country-specific VW availabilities obtained averaging over the period 1996–2005 compare well with the consumption data provided by Hoekstra and Mekonnen^14^ from the same period. Per capita VW availability in 2011 are shown in the Supplementary Material (Figure S2).

The network perturbation is represented by a local crisis inducing a decrease of the VW used for agricultural production in country *x*, which can be caused by environmental or socio-economic events. The decrease of production is expressed as a fraction, *β*, of the initial production value and reads


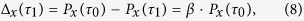


where *τ* is an iterative index and 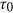 identifies the unperturbed condition. *β* may vary from 0, identifying an absence of perturbation, to 1, identifying an interruption of the country agricultural production (see the following assessments).

The decreased production of agricultural commodities in country *x*, expressed in VW volume, will affect partly the national availability and partly the country exports. The partition between the two terms is chosen to be proportional to the relative weight of availability and export in the country, and the modified terms of the country VW balance read


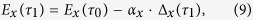



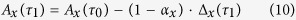


Where 
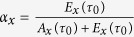
 Parameter 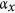 quantifies the *export propensity* of each country and tunes the impact of the perturbation 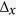 on the network varying within the range [0,1], where 0 identifies a disturbance completely absorbed by a decrease of national availability and 1 identifies a disturbance totally reflected into a reduction of exports, preserving the national internal availability. In the following, a data-based evaluation of such parameter is shown.

The reduction of export (Eq. (9)) is propagated towards all trading partners of country *x* proportionally to the existing flows towards each of these countries. Following Eq. (7), the VW trade from country *x* to all partners, *k*, thus becomes


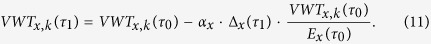


All trading partners will find themselves with a reduced volume of VW in the balance, similarly to country *x* but due to a reduced import rather than a reduced production. The disturbance affecting each importing country is then


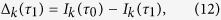


which, assuming that production does not change, will have to be partitioned between internal availability and export according to Eqs (9,10), where 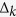 substitutes 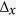 and 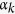 substitutes 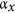. Export reduction is again propagated towards all trading partners and the procedure is repeated iteratively, reproducing the propagation occurring in the network as a result of the initial crisis in country *x*. The recursive expression of the VW balance terms for country *c* at the *n*-th iteration 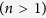 reads


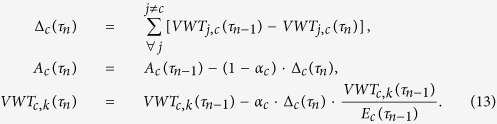


Iterations are interrupted when results reach a prescribed precision, that is when all terms in the trade matrix change less than a fraction *ε* of the previous values, i.e.


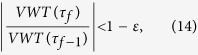


where 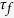 is the final iteration step and *ε* is here taken equal to 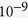. At the end of the simulation, the reduction of production occurred in country *x* reflects into a reduction of VW availability in all countries, satisfying the relation


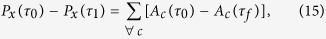


with a geographic distribution of reductions that depends on the trade network. The total iteration time 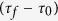 is assumed to be shorter than the temporal scale of data, which is annual, because simulations mimic the continuous adjustments of trade to meet the equilibrium conditions between demand and supply. It is worth noticing that all variations of agricultural production and trade are referred to overall VW volumes, without tracking the composition in terms of commodities.

### Model assumptions and parameter calibration

The propagation model bases on some assumptions and calibrations which are here supported by specific statistical analyses. These analyses are based on the variations of VW import, 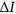, VW export, 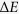, and VW in agricultural production, 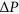, measured in subsequent years within the period 1986–2011 (1961–2011 when only *P* is considered) in all world countries with population greater than 1 million people.

### Variations of export vs. production

The model assumes that a decrease of agricultural production in a country induces a decrease of export in the same country. Such hypothesis has been verified with a sign test considering the sample of 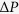 and the corresponding (same country, same years) sample of 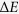 values. The one-tail sign test confirms that when the production decreases (increases), that is 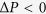 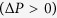, the median of 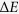 is significantly lower (greater) than zero, with test *p*-value equal to 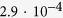. This supports the model assumption that a decrease of production is associated to a decrease of export.

### Variations of export vs. import

Similarly, the model assumes that a decrease of import in a country is related, to different extents, to a decrease of export in the same country. The sign test is thus applied to the sample of 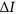 values and the corresponding (same country, same years) 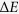 values; the test confirms that when the import decreases (increases), that is 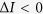 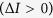, the median of 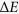 is significantly lower (greater) than zero, with test *p*-value equal to 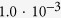. This supports the model assumption that a decrease of import is associated to a decrease of export.

### Variations of import vs. production

Implicitly, the model assumes that a decrease of production induces a decrease of export, rather than an increase of import. A sign test is then applied to the sample of 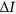 values and the corresponding (all countries, all years) 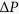 values. Again applying the one-tail test, one finds that when the production decreases (increases), that is 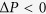 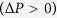, the median of 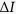 is not significantly lower (greater) than zero, with test *p*-value equal to 0.84. This supports the model assumption that a decrease of production is not generally associated to an increase of import.

### Calibration of the parameter *β*

**Figure 7:**
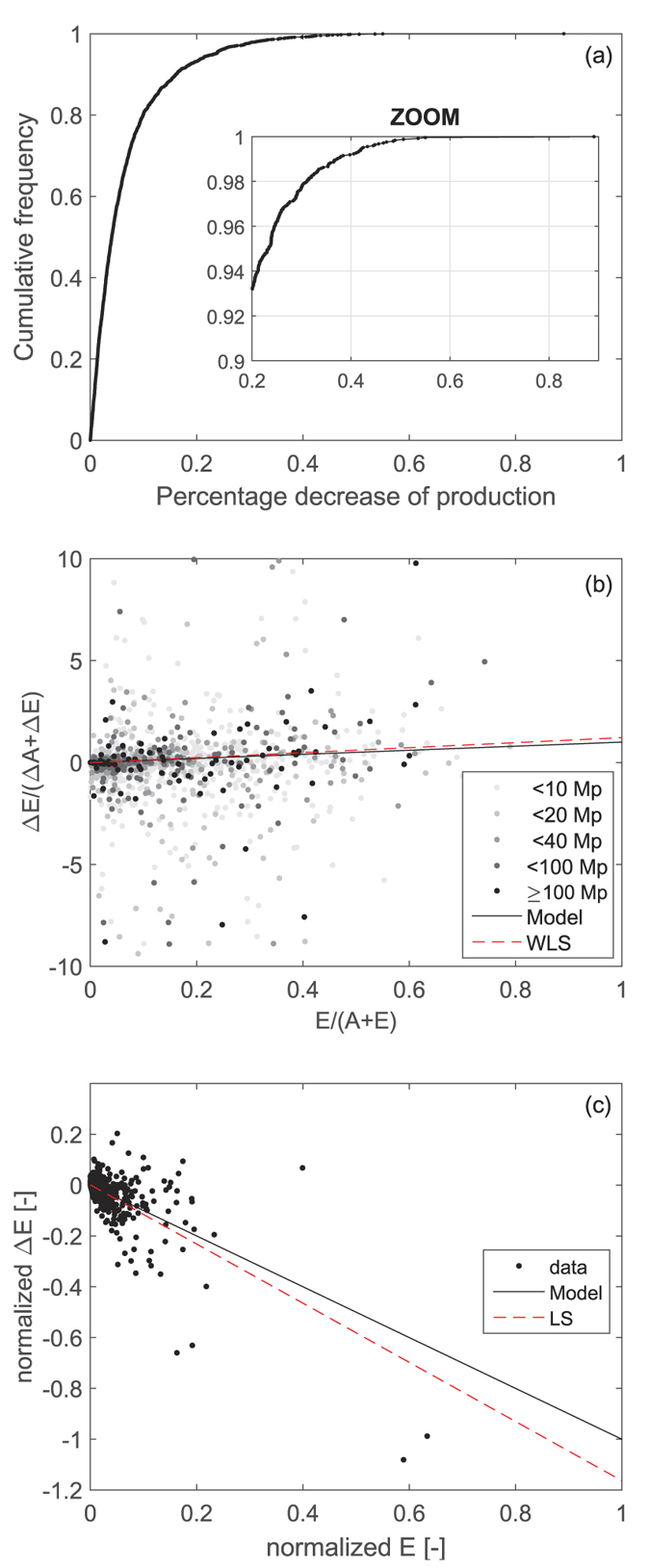
Data-based model calibration (**a**) Cumulative empirical distribution of percentage decreases of VW production in the period 1961–2011. (**b**) Fitting of the partition of crises proportional to the relative fraction of VW export and availability (identified by the 1:1 line), with grey levels scaling with country population, and comparison with a least square linear fitting weighted by country population. (**c**) Fitting of the propagation of crises towards trading partners proportionally to the existing VW fluxes (1:1 line) and comparison with a least square linear fitting.

The *β* parameter represents the entity of a crisis and equals the percentage variation of the VW in agricultural production of the hit country. An empirical distribution function is built for the negative percentage variations of the VW production recorded in all countries and all years from 1961 to 2011 (time span of production data) to guarantee a better definition of the curve tail (see Fig. 7a). The parameter value, 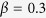, used in the Result section represents a critical event with 2.3% of exceedance relative frequency among all recorded decreases, thus a rare but not unlikely crisis. Considering all years and countries without conditioning to decreases of production, such crisis has an exceedance relative frequency of 0.8% thus, on average, every year one country experiences a similar event, if i.i.d. events are considered.

### Partition of the crisis within each country

Both a decrease of VW in agricultural production (in the country hit by the crisis) and of VW import (in all countries reached by the crisis propagation) determine a decrease of VW volume in the balance of the country (Eq. (1)). This decrease, measured generically with 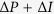, needs to be partitioned between a variation of internal VW availability and a variation of country VW export, provided that 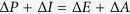, according to Eq. (1). The partition is expressed by the country-specific parameter *α*, or country propensity for export. The parameter is assumed to be proportional to the relative weight of availability and export before the crisis (Eqs (9,10)), i.e.,


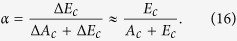


Figure 7b shows the ratio of VW export variations over the summed decrease of internal availability and export (increases are left out) as a function of the country- and year-specific *α* values. Points are depicted with different grey levels according to the population of countries they refer to, to differentiate between large and small countries.

Points are rather scattered due to the variety of country conditions and behaviors. The 1:1 line represents the model assumption that the partition of crises within countries depends on their export propensity (Eqs (9,10)). Although a common pattern does not emerge in country behaviors, the proposed model fits reasonably well with most points and in particular with those corresponding to large countries. The least-square regression line, weighted by country populations (dashed red line in Fig. 7b), is found very close to the model line, supporting the model assumption.

It may also be noticed that several cases exist in which countries increase their exports despite a reduction of summed internal availability and export (negative vertical axis). In such cases, losses are compensated by the reduction of VW availability and likely by stocks alone, which are mainly devoted to compensate for variable/uncertain food availability.

### Propagation of the crisis towards the partners

The model assumes that VW export variations of a country are propagated towards trading partners proportionally to the virtual water flows exchanged before the crisis. This has been assessed on a sample of cases identified by a decrease of country VW export larger than 10 km^3^ from one year to the following, provided that such decrease represented at least 10% of the initial export. This identified 20 cases in 26 years. In each case, initial fluxes towards the trading partners were normalized by the total VW export and the variations of flux were normalized with respect to the total variation of export. These values are then plotted in Fig. 7c, where the model rule of proportionality is represented by a single line (red solid line) and it compares well with data (coefficient of determination, 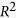, equal to 0.501) and with the least-square linear regression fitted to data. The same analysis performed with a less restrictive threshold of export decrease (1 km^3^) identifies 226 cases; the model fitting is still very good 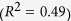.

The propagation of an export decrease among trade partners has been investigated using also other variables than the initial VW export flux. Both direct and inverse relationships have been sought with destination-country per-capita GDP, total GDP and population; however, none of these variables explained globally or locally more than 10% of the variance of flux variations 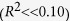.

### Indicators of impact and vulnerability

The proposed propagation model allows one to distribute an initial local perturbation to the whole network accounting for the network topology and virtual water flows. The procedure can be repeated applying the same initial perturbation to each of the *N* countries, obtaining a matrix of resulting internal availability in country *c*, 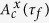, due to a perturbation in country *x*. Notice the addition of the apex *x* to identify the country hit by the crisis.

Some indicators are then useful to investigate the effects of local crises in the virtual water trade network. The absolute impact of a perturbation occurring in country *x* can be expressed through Eq. 2, where 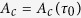 and 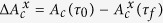, and it depends on the country VW exports and the VW volume losses induced by the crisis. The normalized impact divides 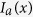 by the uniform percentage variation of VW availability induced in the world by the crisis, excluding the country of origin (Eq. (3)). Such normalized impact, 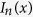, is independent on the crisis entity and it takes identical values if -for example- crises are defined by a given variation of VW export, rather than the loss of a given fraction of agricultural production. This normalized impact highlights the role of the network in defining the effects of crises. This is because it differentiates crises according to the most/least affected countries, taking values close to (or different from the) unit if countries are affected proportionally (or not proportionally) to their VW internal availability. Both absolute impact and normalized impact values are quantified for all countries in the Supplementary Table.

Measures of impact characterize the countries of origin of the crises, thus their active role in the propagation, as opposed to the passive role with respect to crises originated elsewhere, described by the country vulnerability. Vulnerability can be measured by the average percentage reduction of VW internal availability induced in country *c* by the perturbations triggered in all other countries, (Eq. (4)). In this case no population weight is necessary, as the percentage reduction within the summation term are referred to the same country.

The world-average population-weighted formulation of the variables (normalized impact and vulnerability), in a given year, are


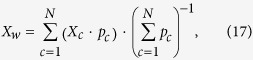


where 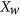 is the world average value, 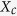 are the country-specific values of the considered variable and 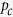 is the country population. The sum is extended to all countries in the world, *N*, that are active in the considered year.

## Additional Information

**How to cite this article**: Tamea, S. *et al.* Global effects of local food-production crises: a virtual water perspective. *Sci. Rep.* **6**, 18803; doi: 10.1038/srep18803 (2016).

## Supplementary Material

Supplementary Information

## Figures and Tables

**Table 1:** Numerical details on the crises shown in Fig. 2: entity of the crisis, characteristics of the hit node and variations measured in the major trading partner (Δ*I*: variation of VW import, Δ*A*_*pc*_: variation of per-capita internal availability, Δ*A*_*r*_: percentage variation of per-capita internal availability).

Crisis in:	USA	Italy	Japan	South Africa
total VW loss [km^3^]	317.7	24.3	14.7	25.5
hit-node unweighted degree [-]	373	361	276	331
hit-node weighted degree [km^3^]	511.4	115.4	110.7	32.8
hit-node eigenvector centrality [-]	0.566	0.042	0.003	0.012
major partner in 2011	China	Germany	Hong Kong	Mexico
Δ*I* in major partner [km^3^]	27.45	0.917	0.020	0.467
Δ*A*_*pc*_ in major partner [m^3^/p]	19.7	7.2	1.2	3.5
Δ*A*_*r*_ in major partner [%]	1.21	0.42	0.08	0.17

## References

[b1] Allan J. Virtual water: a strategic resource global solutions to regional deficits. Groundwater 36, 545–546 (1998).

[b2] Reimer J. On the economics of virtual water trade. Ecol. Econ. 75, 135–139 (2012).

[b3] Hoekstra A. & Chapagain A. Water footprints of nations: water use by people as a function of their consumption pattern. Water Resour. Managem. 21, 35–48 (2007).

[b4] Allan J. Fortunately there are substitutes for water otherwise our hydro-political futures would be impossible. In Priorities for water resources allocation and management 1–21 (Overseas Development Administration, London, UK, 1993).

[b5] FAO. Coping with water scarcity - An action framework for agriculture and food security. Tech. Rep. Water Report n. 38, Food and Agriculture Organization of the United Nations (2012).

[b6] D’Odorico P. & Rulli M. The fourth food revolution. Nature Geoscience 6, 417–418 (2013).

[b7] De Fraiture C., Cai X., Amarasinghe U., Rosegrant M. & Molden D. Does international cereal trade save water? The impact of virtual water tarde on global water use. Tech. Rep. Comprehensive Assessment Research Report n. 4, Int. Water Manag. Inst., Colombo (2004).

[b8] Chapagain A., Hoekstra A. & Savenije H. Water saving through international trade of agricultural products. Hydrol. Earth Syst. Sci. 10, 455–468 (2006).

[b9] Wichelns D. Virtual water: a helpful perspective, but not a sufficient policy criterion. Water Resour. Manage. 24, 2203–2219 (2010).

[b10] Seekell D., D’Odorico P. & Pace M. Virtual water transfers unlikely to redress inequality in global water use. Environ. Res. Lett. 6, 024017 (2011).

[b11] Garlaschelli D. & Loffredo M. Structure and evolution of the world trade network. Physica A: Statistical Mechanics and its Applications 355, 138–144 (2005).

[b12] Fagiolo G., Reyes J. & Schiavo S. The evolution of the world trade web: a weighted-network analysis. J. Evol. Econ. 20, 479–514 (2010).

[b13] Ercsey-Ravasz M., Toroczkai Z., Lakner Z. & Baranyi J. Complexity of the international agro-food trade network and its impact on food safety. PLoS ONE 7, e37810 (2012). 22701535 10.1371/journal.pone.0037810 PMC3365103

[b14] Hoekstra A. & Mekonnen M. The water footprint of humanity. Proc. Natl. Acad. Sci. 109, 3232–3237 (2012). 22331890 10.1073/pnas.1109936109 PMC3295316

[b15] Carr J., D’Odorico P., Laio F. & Ridolfi L. On the temporal variability of the virtual water network. Geophys. Res. Lett. 39, L06404 (2012).

[b16] Konar M., Dalin C., Hanasaki N., Rinaldo A. & Rodriguez-Iturbe I. Temporal dynamics of blue and green virtual water trade networks. Water Resour. Res. 48, W07509 (2012). 10.1073/pnas.1203176109 PMC3341016 22474363

[b17] Dalin C., Konar M., Hanasaki N., Rinaldo A. & Rodriguez-Iturbe I. Evolution of the global virtual water trade network. Proc. Natl. Acad. Sci. 109, 5989–5994 (2012). 22474363 10.1073/pnas.1203176109 PMC3341016

[b18] Suweis S., Rinaldo A., Maritan A. & D’Odorico P. Water-controlled wealth of nations. Proc. Natl. Acad. Sci. 110, 4230–4233 (2013). 23359709 10.1073/pnas.1222452110 PMC3600477

[b19] D’Odorico P., Laio F. & Ridolfi L. Does globalization of water reduce societal resilience to drought? Geophys. Res. Lett. 37, L13403 (2010).

[b20] Falkenmark M. & Rockström J. Balancing water for humans and nature: the new approach in Ecohydrology (Earthscan, London, UK, 2004).

[b21] Hanjra M. & Qureshi M. Global water crisis and future security in an era of climate change. Food Policy 35, 365–377 (2010).

[b22] Porkka M., Kummu M., Siebert S. & Varis O. From food insufficiency towards trade dependency: a historical analysis of global food availability. PLoS ONE 8, e82714 (2013). 24367545 10.1371/journal.pone.0082714 PMC3867377

[b23] Helbing D. Globally networked risks and how to respond. Nature 497, 51–59 (2013). 23636396 10.1038/nature12047

[b24] Bollobás B. Random graphs (Cambridge University Press, Cambridge, UK, 2001). 2nd Edition.

[b25] Battiston S., Delli Gatti D., Gallegati M., Greenwald B. & Stiglitz J. Liaisons dangereuses: increasing connectivity, risk sharing, and systemic risk. J. Econ. Dyn. Control 36, 1121–1141 (2012).

[b26] Gai P. & Kapadia S. Contagion in financial networks. Proc. R. Soc. A 466, 2401–2423 (2010).

[b27] Allen F. & Gale D. Financial contagion. J. Polit. Econ. 108, 1–33 (2000).

[b28] Fagiolo G. & Alatriste Contreras M. Propagation of economic shocks in Input-Output networks: a cross-country analysis (2014). ArXiv: 1401.4704v2. 10.1103/PhysRevE.90.062812 25615153

[b29] Acemoglu D., Carvalho V., Ozdaglar A. & Tahbaz-Salehi A. The network origins of aggregate fluctuations. Econometrica 80, 1977–2016 (2012).

[b30] Serrano M. & Boguna M. Topology of the world trade web. Phys. Rev. E 68, 015101 (2003). 10.1103/PhysRevE.68.015101 12935184

[b31] Lee K.-M. *et al.* Impact of the topology of global macroeconomic network on the spreading of economic crises. PLoS ONE 6, e18443 (2011). 21483794 10.1371/journal.pone.0018443 PMC3069097

[b32] Kali R. & Reyes J. Financial contagion on the international trade network. Econ. Inq. 48, 1072–1101 (2010).

[b33] Foti N., Pauls S. & Rockmore D. Stability of the world trade web over time - An extinction analysis. J. Econ. Dyn. Control 37, 1889–1910 (2013).

[b34] Berrittella M., Hoekstra A., Rehdanz K., Roson R. & Tol R. The economic impact of restricted water supply: a computable general equilibrium analysis. Water Research 41, 1799–1813 (2007). 17343892 10.1016/j.watres.2007.01.010

[b35] Calzadilla A., Rehdanz K. & Tol R. The economic impact of more sustainable water use in agriculture: a computable general equilibrium analysis. J. Hydrol. 384, 292–305 (2010).

[b36] BBC News. Argentine MPs approve farm taxes. Available at http://news.bbc.co.uk/2/hi/americas/7491725.stm (2008, July 5^*th*^). Accessed on 25/08/2015.

[b37] CNN International. Store shelves grow bare as Argentine farmers continue strike. Available at http://edition.cnn.com/2008/WORLD/americas/03/25/argentina.strike/index.html (2008, March 26^*th*^). Accessed on 25/08/2015.

[b38] IPS (Inter Press Service). Agriculture-Argentina: worst drought in 100 years. Available at http://www.ipsnews.net/2009/01/agriculture-argentina-worst-drought-in-100-years/ (2009, January 21^*st*^). Accessed on 25/08/2015.

[b39] The Economist. Food and the arab spring - Let them eat baklava. Available at http://www.economist.com/node/21550328 (2012, March 17^*th*^). Accessed on 25/08/2015.

[b40] Newman M. Networks: an introduction (Oxford University Press, New York, USA, 2010).

[b41] Antonelli M. & Tamea S. Food-water security and virtual water trade in the middle east and north africa. Int. J. Water Resour. Dev. 31, 326–342 (2015).

[b42] Hoff H. *et al.* Greening the global water system. J. Hydrol. 384, 177–186 (2010).

[b43] FAO. Review of world water resources by country. Tech. Rep. Water Report n. 23, Food and Agriculture Organization of the United Nations (2003).

[b44] FAO, Statistics Division. FAOSTAT online database. Available at http://faostat3.fao.org/home/index.html (2013). Accessed on 25/9/2013.

[b45] Mekonnen M. & Hoekstra A. The green, blue and grey water footprint of crops and derived crop products. Tech. Rep. Value of Water Research Report Series n. 47, UNESCO-IHE, Delft, The Netherlands (2010).

[b46] Mekonnen M. & Hoekstra A. The green, blue and grey water footprint of farm animals and animal products. Tech. Rep. Value of Water Research Report Series n. 48, UNESCO-IHE, Delft, The Netherlands (2010).

[b47] Tamea S. *et al.* Local and global perspectives on the virtual water trade. Hydrol. Earth Syst. Sci. 17, 1205–1215 (2013).

[b48] FAO, Land and Water Division. AQUASTAT online database. Available at http://www.fao.org/nr/water/aquastat/main/index.stm (2014). Accessed on 20/10/2014.

[b49] United Nations. National accounts online database. Available at http://unstats.un.org/unsd/snaama/dnllist.asp (2014). Accessed on 29/10/2014.

