# Corrigendum: Global effects of local food-production crises: a virtual water perspective

**DOI:** 10.1038/srep46987

**Published:** 2018-05-22

**Authors:** Stefania Tamea, Francesco Laio, Luca Ridolfi

Scientific Reports 6: Article number: 1880310.1038/srep18803; published online: 01 25 2016; updated: 05 22 2018

The Acknowledgements section in this Article is incomplete.

“The authors acknowledge funding from the Italian Ministry of Education, University and Research (MIUR) through the project “The global virtual-water network: social, economic, and environmental implications” (FIRB - RBFR12BA3Y).”

should read:

“The authors acknowledge funding from the Italian Ministry of Education, University and Research (MIUR) through the project “The global virtual-water network: social, economic, and environmental implications” (FIRB - RBFR12BA3Y). Funding from the European Research Council (ERC) is also acknowledged (CWASI Project, grant #647473).”

