# Supplementary material for: Global effects of local food-production crises: a virtual water perspective
Source: Sci Rep. 2016 Jan 25;6:18803. doi: 10.1038/srep18803 (PMC4726167; doi:10.1038/srep18803)
Supplement: Supplementary Information [file srep18803-s1.pdf]

Global effects of local food-production crises: a virtual water perspective

Stefania Tamea[1,*], Francesco Laio[1], and Luca Ridolfi[1]

Politecnico di Torino, Dept. of Environment, Land and Infrastructure Engineering, Turin, Italy
(*) stefania.tamea@polito.it

SUPPLEMENTARY INFORMATION

All figures are authors' elaborations with Matlab® R14 software and maps are created with the Mapping Toolbox, v.2.0.3 (*http://uk.mathworks.com/products/mapping/*).

Temporal trends of virtual water in Argentina

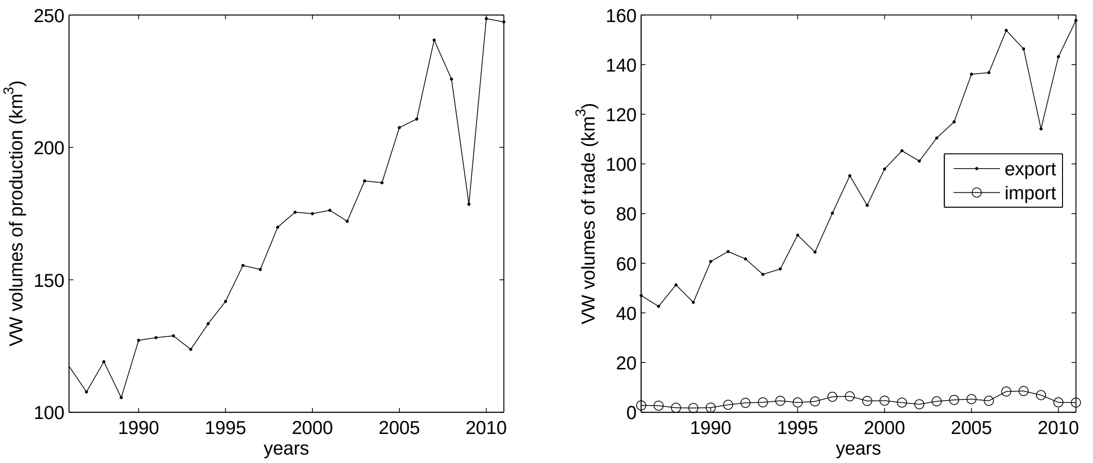

Figure S1: Virtual water volumes embedded in agricultural primary production (left) and in trade (right) of Argentina in different years (data are expressed in km^3).

Identification of high-consuming and low-consuming countries in 2011

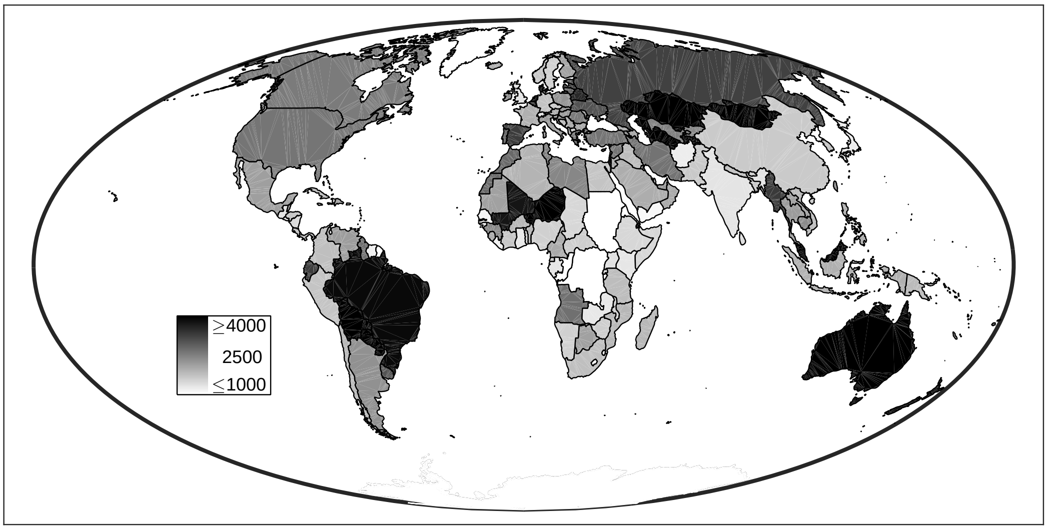

Figure S2: Per capita volume of virtual water associated to the internal availability of food (food supply + stock) in each country in 2011 (in m^3/person).

Impact versus vulnerability as a function of countries' wealth

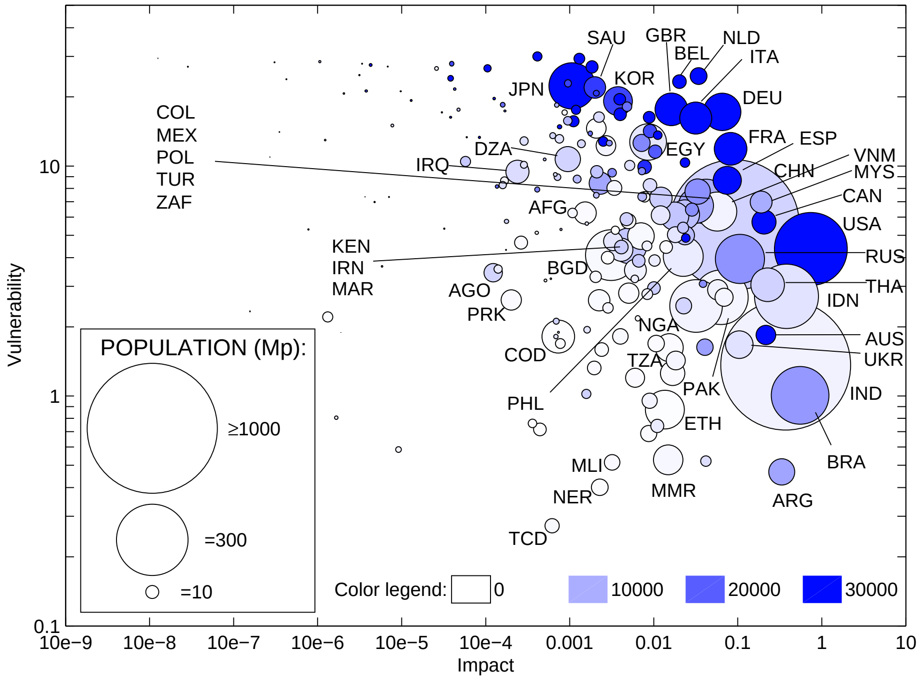

Figure S3: Absolute impact versus vulnerability of world countries with circle sizes indicating country populations (in million people) and colors scaled according to the per-capita GDP of countries (in US dollars/person/year).

Temporal variation of indexes by country

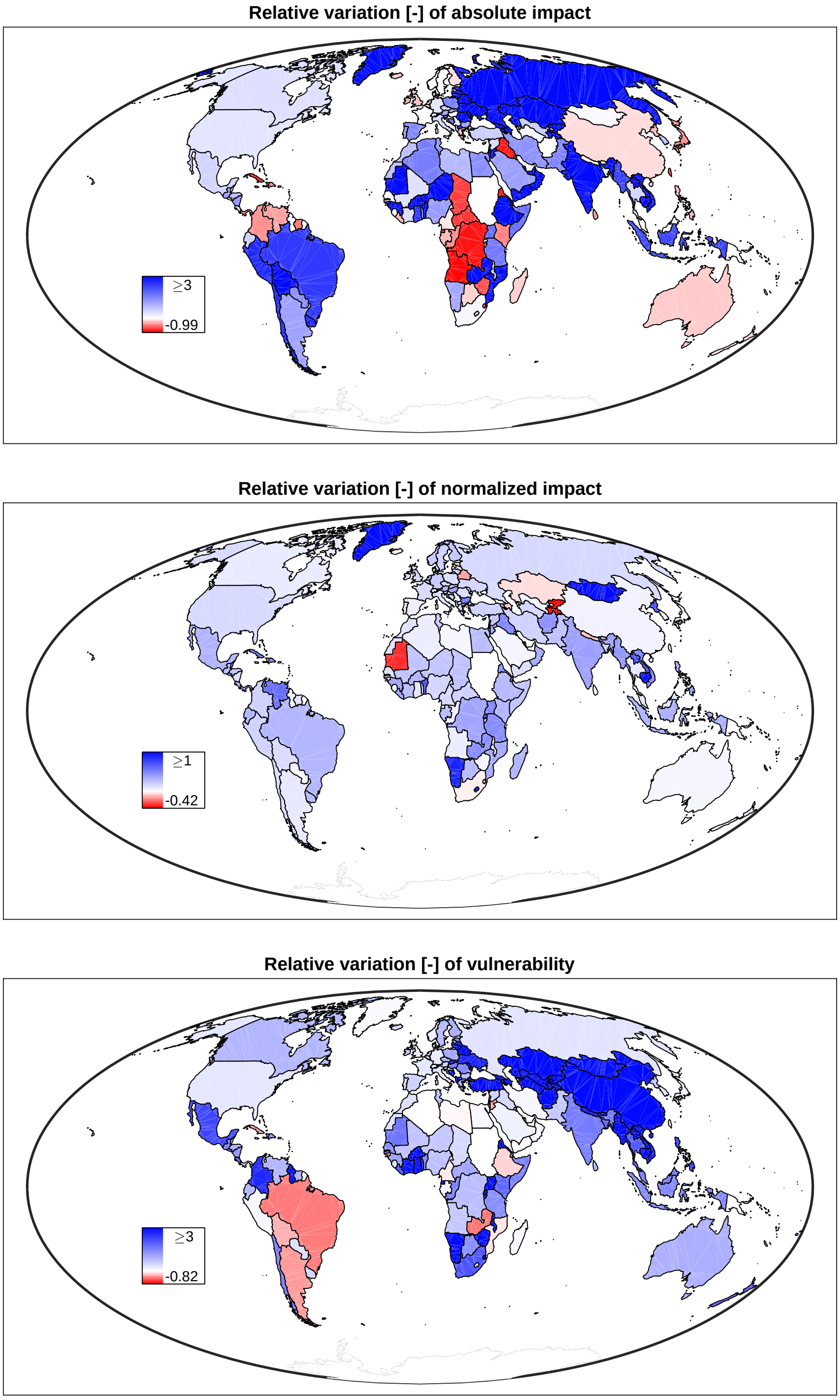

Figure S4: Relative variation of absolute impact (above), normalized impact (middle) and vulnerability (below) by country, from 1986 (or the first year of country activity) to 2011.

Impact and vulnerability of countries

Table S1: Values of absolute impact, I_a, normalized impact, I_n, and vulnerability V of all countries in 2011.

**Country**	**Country code**	I_a	I_n	V
Afghanistan	AFG	1.53e-003	1.037	6.05
Albania	ALB	1.59e-004	0.914	8.17
Algeria	DZA	9.50e-004	0.846	10.32
American Samoa	ASM	7.08e-006	1.247	7.32
Angola	AGO	1.23e-004	0.694	3.34
Antigua and Barbuda	ATG	9.74e-006	1.062	21.09
Argentina	ARG	3.35e-001	0.954	0.45
Armenia	ARM	2.85e-004	0.891	10.02
Australia	AUS	2.17e-001	1.088	1.79
Austria	AUT	8.87e-003	0.961	16.01
Azerbaijan	AZE	2.10e-003	0.690	9.26
Bahamas	BHS	3.79e-006	0.992	21.12
Bahrain	BHR	3.95e-005	1.082	27.68
Bangladesh	BGD	3.11e-003	1.310	3.80
Barbados	BRB	1.69e-004	1.461	17.30
Belarus	BLR	6.63e-003	0.647	3.80
Belgium	BEL	2.02e-002	0.957	22.82
Belize	BLZ	7.89e-004	1.138	5.28
Benin	BEN	1.41e-002	1.367	4.36
Bermuda	BMU	3.38e-006	1.132	27.79
Bhutan	BTN	1.67e-006	0.940	0.80
Bolivia	BOL	1.11e-002	0.953	0.73
Bosnia and Herzegovina	BIH	1.38e-003	0.943	12.36
Botswana	BWA	2.08e-003	1.163	7.41
Brazil	BRA	5.52e-001	0.969	0.92
Brunei Darussalam	BRN	1.26e-004	1.125	19.57
Bulgaria	BGR	2.24e-002	0.821	5.30
Burkina Faso	BFA	1.07e-002	1.058	1.65
Burundi	BDI	4.42e-004	1.063	0.70
Cambodia	KHM	4.02e-003	0.990	1.77
Cameroon	CMR	1.83e-002	0.851	1.39
Canada	CAN	2.06e-001	1.002	5.51
Cape Verde	CPV	7.74e-006	1.214	14.95
Cayman Islands	CYM	3.07e-007	0.812	28.24
CAR	CAF	3.62e-004	1.003	0.75
Chad	TCD	6.19e-004	1.002	0.27
Chile	CHL	7.18e-003	0.952	12.29
Hong Kong	HKG	1.31e-003	1.083	28.83
Macao	MAC	4.29e-006	1.165	27.38
China (mainland)	CHN	9.15e-002	1.120	4.57
Taiwan	TWN	2.08e-003	1.096	14.15
Colombia	COL	1.78e-002	0.965	5.81
Comoros	COM	4.05e-004	0.989	5.10
... (continue) ...				

**Country**	**Country code**	I_a	I_n	V
... (continue) ...				
Congo	COG	2.87e-004	0.842	12.69
Congo DR	COD	7.34e-004	1.075	1.73
Cook Islands	COK	3.51e-007	1.267	14.06
Costa Rica	CRI	7.32e-003	0.980	7.26
Cote d'Ivoire	CIV	6.91e-002	0.948	2.62
Croatia	HRV	3.20e-003	0.981	9.24
Cuba	CUB	4.13e-003	1.038	4.35
Cyprus	CYP	7.58e-004	0.846	14.71
Czech Republic	CZE	1.04e-002	0.938	11.32
Denmark	DNK	2.36e-002	1.048	10.20
Djibouti	DJI	2.60e-005	1.018	26.44
Dominica	DMA	1.75e-004	1.417	4.31
Dominican Republic	DOM	4.77e-003	0.875	5.76
Ecuador	ECU	2.28e-002	0.910	2.41
Egypt	EGY	8.58e-003	1.032	12.04
El Salvador	SLV	5.19e-003	1.026	9.91
Equatorial Guinea	GNQ	1.36e-004	0.669	8.10
Eritrea	ERI	1.32e-006	1.626	2.18
Estonia	EST	1.76e-003	0.798	13.76
Ethiopia	ETH	1.36e-002	1.036	0.82
Falkland Islands	FLK	1.18e-005	1.191	8.91
Faroe Islands	FRO	2.86e-008	0.910	26.99
Fiji	FJI	6.74e-004	1.121	9.14
Finland	FIN	2.52e-003	0.889	12.60
France	FRA	8.26e-002	0.925	11.31
French Polynesia	PYF	3.83e-005	0.949	16.24
Gabon	GAB	4.12e-004	0.857	7.85
Gambia	GMB	8.71e-004	1.367	16.97
Georgia	GEO	7.57e-004	0.772	12.97
Germany	DEU	6.55e-002	0.897	16.26
Ghana	GHA	5.81e-002	0.966	2.81
Greece	GRC	7.81e-003	0.929	9.70
Greenland	GRL	6.83e-006	1.245	27.05
Grenada	GRD	3.02e-005	0.938	17.10
Guam	GUM	1.23e-006	0.859	12.94
Guatemala	GTM	1.81e-002	0.997	4.91
Guinea	GIN	1.97e-003	1.046	1.30
Guinea-Bissau	GNB	6.47e-003	1.366	2.16
Guyana	GUY	1.60e-003	1.079	5.59
Haiti	HTI	2.65e-004	0.858	4.56
Honduras	HND	1.03e-002	1.010	3.82
Hungary	HUN	2.86e-002	0.902	6.34
Iceland	ISL	8.43e-005	0.969	13.26
India	IND	3.74e-001	1.136	1.12
Indonesia	IDN	3.81e-001	1.033	2.47
Iran	IRN	5.07e-003	0.991	4.27
Iraq	IRQ	2.39e-004	1.204	9.12
... (continue) ...				

Country	Country code	I_a	I_n	V
... (continue) ...				
Ireland	IRL	1.12e-002	1.164	13.44
Israel	ISR	1.11e-003	0.920	15.40
Italy	ITA	3.17e-002	0.967	15.42
Jamaica	JAM	7.19e-004	1.120	8.85
Japan	JPN	1.07e-003	1.049	20.81
Jordan	JOR	2.23e-003	1.073	16.06
Kazakhstan	KAZ	4.08e-002	0.830	1.59
Kenya	KEN	7.07e-003	1.119	4.76
Kiribati	KIR	3.22e-005	0.817	5.33
Korea DPR	PRK	2.03e-004	1.183	2.53
Korea R	KOR	3.76e-003	1.201	18.37
Kuwait	KWT	1.05e-004	1.008	26.34
Kyrgyzstan	KGZ	1.08e-003	0.696	6.16
Laos	LAO	2.87e-003	1.116	2.38
Latvia	LVA	2.96e-003	0.800	12.47
Lebanon	LBN	9.52e-004	0.992	15.53
Lesotho	LSO	9.20e-006	1.391	0.58
Liberia	LBR	3.49e-003	0.953	5.19
Libya	LBY	5.76e-005	0.855	10.32
Lithuania	LTU	7.24e-003	0.793	9.40
Macedonia	MKD	6.29e-004	0.923	13.44
Madagascar	MDG	6.01e-003	1.019	1.17
Malawi	MWI	8.72e-003	1.203	0.67
Malaysia	MYS	1.90e-001	1.049	6.75
Maldives	MDV	1.06e-006	1.417	28.33
Mali	MLI	3.20e-003	1.101	0.50
Malta	MLT	4.29e-005	0.992	21.53
Marshall Islands	MHL	1.57e-007	0.805	2.33
Mauritania	MRT	1.67e-004	0.763	8.53
Mauritius	MUS	7.02e-004	1.003	18.29
Mayotte	MYT	0.00e+000	NaN	30.00
Mexico	MEX	4.20e-002	0.891	6.55
Micronesia	FSM	7.76e-007	0.655	5.29
Moldova	MDA	5.96e-003	0.802	3.19
Mongolia	MNG	1.62e-003	0.885	1.92
Montenegro	MNE	4.75e-005	0.976	17.51
Montserrat	MSR	1.89e-006	0.899	1.89
Morocco	MAR	4.13e-003	0.898	4.17
Mozambique	MOZ	5.07e-003	1.054	2.72
Myanmar	MMR	1.49e-002	1.145	0.50
Namibia	NAM	6.94e-004	1.133	2.10
Nauru	NRU	2.71e-007	1.028	10.94
Nepal	NPL	2.24e-003	1.326	2.53
Netherlands	NLD	3.43e-002	0.987	24.00
Netherlands Antilles	ANT	-2.03e-007	1.200	30.11
New Caledonia	NCL	1.30e-005	1.195	19.20
New Zealand	NZL	2.42e-002	0.997	4.80
... (continue) ...				

Country	Country code	I_a	I_n	V
... (continue) ...				
Nicaragua	NIC	8.34e-003	1.001	4.42
Niger	NER	2.28e-003	1.149	0.39
Nigeria	NGA	3.20e-002	0.997	2.28
Niue	NIU	2.63e-007	0.781	0.57
Norway	NOR	1.19e-003	1.032	17.33
Palestine	PSE	1.41e-004	0.962	3.52
Oman	OMN	9.58e-004	1.099	22.65
Pakistan	PAK	6.31e-002	1.185	2.48
Palau	PLW	0.00e+000	NaN	30.00
Panama	PAN	1.23e-003	0.944	8.68
Papua New Guinea	PNG	8.39e-003	0.900	2.73
Paraguay	PRY	4.19e-002	0.859	0.51
Peru	PER	1.22e-002	0.950	7.00
Philippines	PHL	2.25e-002	1.013	3.81
Poland	POL	3.33e-002	0.940	7.46
Portugal	PRT	9.06e-003	0.784	13.91
Qatar	QAT	3.83e-005	1.228	23.90
Romania	ROU	2.36e-002	0.876	4.85
Russian Federation	RUS	1.06e-001	0.890	3.66
Rwanda	RWA	2.41e-003	1.490	1.56
Saint Helena	SHN	0.00e+000	NaN	30.00
Saint Kitts and Nevis	KNA	4.24e-007	1.127	23.81
Saint Lucia	LCA	2.06e-006	1.020	20.62
Saint Pierre and Miquelon	SPM	1.26e-008	0.973	29.42
Saint Vincent and the Grenadines	VCT	5.97e-005	1.080	13.32
Samoa	WSM	5.87e-006	0.805	3.65
Sao Tome and Principe	STP	5.98e-004	0.769	3.23
Saudi Arabia	SAU	2.00e-003	1.122	21.26
Senegal	SEN	3.40e-003	0.914	7.85
Serbia	SRB	1.01e-002	0.928	2.89
Seychelles	SYC	3.14e-006	0.835	24.81
Sierra Leone	SLE	7.76e-004	0.813	1.66
Singapore	SGP	4.14e-004	1.034	29.52
Slovakia	SVK	4.83e-003	0.954	17.87
Slovenia	SVN	2.07e-003	0.913	20.54
Solomon Islands	SLB	5.17e-004	0.854	3.16
Somalia	SOM	2.84e-003	1.055	3.92
South Africa	ZAF	2.35e-002	1.054	5.88
Spain	ESP	7.51e-002	0.919	8.31
Sri Lanka	LKA	1.21e-002	0.964	5.94
Suriname	SUR	5.05e-004	0.899	10.64
Swaziland	SWZ	6.86e-004	0.869	1.80
Sweden	SWE	4.00e-003	0.900	16.50
Switzerland	CHE	3.94e-003	0.869	19.19
Syria	SYR	3.29e-003	0.973	4.58
Tajikistan	TJK	2.04e-003	0.508	3.24
Tanzania	TZA	1.54e-002	1.286	1.56
... (continue) ...				

**Country**	**Country code**	I_a	I_n	V
... (continue) ...				
Thailand	THA	2.27e-001	1.071	2.90
Timor-Leste	TLS	1.77e-004	1.021	5.71
Togo	TGO	7.80e-003	0.930	7.23
Tonga	TON	4.76e-006	0.893	6.96
Trinidad and Tobago	TTO	1.59e-004	1.013	18.36
Tunisia	TUN	9.03e-003	0.850	8.08
Turkey	TUR	3.19e-002	0.994	6.38
Turkmenistan	TKM	1.58e-003	0.883	1.01
Tuvalu	TUV	3.68e-006	1.261	7.34
Uganda	UGA	1.68e-002	1.166	1.21
Ukraine	UKR	1.05e-001	0.864	1.60
UAE	ARE	1.85e-003	1.040	26.50
UK	GBR	1.62e-002	0.896	16.81
USA	USA	7.45e-001	1.066	3.91
Uruguay	URY	3.86e-002	0.992	3.04
Uzbekistan	UZB	6.06e-003	0.830	3.41
Vanuatu	VUT	7.38e-004	0.782	1.89
Venezuela	VEN	2.32e-003	1.164	8.14
Vietnam	VNM	5.72e-002	0.993	5.99
Yemen	YEM	2.71e-003	1.057	11.91
Zambia	ZMB	8.94e-003	1.502	0.93
Zimbabwe	ZWE	5.01e-003	1.047	5.62
